# Facial edema after covid-19 ameliorated by acupuncture: A CARE-compliant case report

**DOI:** 10.1097/MD.0000000000043407

**Published:** 2025-07-18

**Authors:** Yike Han, Shiqiao He, Crystal Lynn Keeler, Hongwu Yin, Lifang Chen

**Affiliations:** a The Third School of Clinical Medicine, Zhejiang Chinese Medical University, Hangzhou, China; b Innovations to Wellness, Oakland, CA; c The Third Affiliated Hospital of Zhejiang Chinese Medical University, Hangzhou City, Zhejiang Province, China.

**Keywords:** acupuncture, case report, covid-19, facial edema

## Abstract

**Rationale:**

Covid-19-induced inflammation and edema of the facial skin is a new problem, albeit still somewhat uncommon. To date, there are no published reports on the use of acupuncture for treating facial edema caused by corona virus disease 2019 (COVID-19).

**Patient concerns:**

The patient, a young female, developed a fever of 38.7 °C, malaise, and headache, followed by facial edema. After 3 months of conventional treatments (i.e., oral hydroxychloroquine and ebastine) failed, she turned to acupuncture.

**Diagnoses:**

Facial edema after COVID-19.

**Interventions:**

Acupuncture points on the face (ST2, ST3, ST4, LI20, and SI18), facial acupuncture with multiple micro-needles in the affected area, and distal acupuncture points on LI11, LI4, LI6, SP9, ST36, ST40, and LR3. The patient was treated with acupuncture in 20 sessions over 2 months (3 times a week for the first month, 2 times a week for the second month) and instructed not to use any medication during and after the treatment of acupuncture.

**Outcomes:**

After 2 months of acupuncture treatment, the facial edema of the patient subsided.

**Lessons:**

This case suggests that acupuncture may be an effective alternative therapy for superficial skin inflammation and edema caused by viral infections like COVID-19. Further studies are warranted to explore acupuncture’s role in managing post-viral inflammatory conditions.

## 1. Introduction

Many patients infected with corona virus disease 2019 (COVID-19) experience varying degrees of different symptoms involving various organ systems throughout the body. The widespread presence of angiotensin converting enzyme 2 (ACE2) receptors in tissues explains 1 mechanism by which COVID-19 is able to impact such a wide variety of areas,^[1]^ with a tendency to attack tissues that were already previously weak or held preexisting inflammation.^[2]^ In addition, high levels of ACE2 are found in keratinocytes and basil cells, indicating a possible route of susceptibility in skin tissue.^[3]^ A significant proportion of COVID-19 survivors continue to have symptoms that persist months after the acute phase of the disease.

Cutaneous symptoms are also observed in severe acute respiratory syndrome coronavirus 2 (SARS-CoV-2) infections. The incidence of skin lesions caused by SARS-CoV-2 has been reported to range from 0.2% to 29%. A variety of skin lesions, including maculopapular rash, urticaria, and blisters, can be observed in patients with COVID-19.^[4]^ Among the above cutaneous manifestations, measles-like changes are the most common manifestation of cutaneous inflammation, with an incidence of 36.1%, often occurring in conjunction with systemic symptoms and correlating with the severity of COVID-19.^[5]^ A handful of case reports found facial swelling in conjunction with infection by COVID-19.^[6–9]^ However, at the time of this submission, no published reports on acupuncture treatment of facial edema caused by COVID-19 could be found. The current mainstay of treatment for long COVID treatment is the use of antihistamines,^[10]^ with some emerging treatments such as hyperbaric oxygen,^[11]^ montelukast,^[12]^ and dexpifenidone.^[13,14]^ One study found a long-term benefit of using human umbilical cord mesenchymal stem cells in the treatment of critically ill patients with neocoronary arthritis for the recovery of lung lesions.^[15]^

Since both the 2003 SARS and 2019 COVID-19 pandemics occurred in China, traditional Chinese medicine and acupuncture are widely used, especially in the sequelae stage. These case studies provide ample clinical information on the use and efficacy of acupuncture.^[16]^ The patient in this case developed facial edema after infection with COVID-19, which did not improve after 3 months of Western medicine treatment. After that time she turned to acupuncture treatment with favorable outcomes.

## 2. Case presentation

This case was reported in accordance with the CARE Checklist guidelines.

This case study ethical approval was obtained from ethics committee of the Third Affiliated Hospital of Zhejiang Chinese Medical University and the patient gave her consent for using her clinical data for this case report.

**Figure 1.**
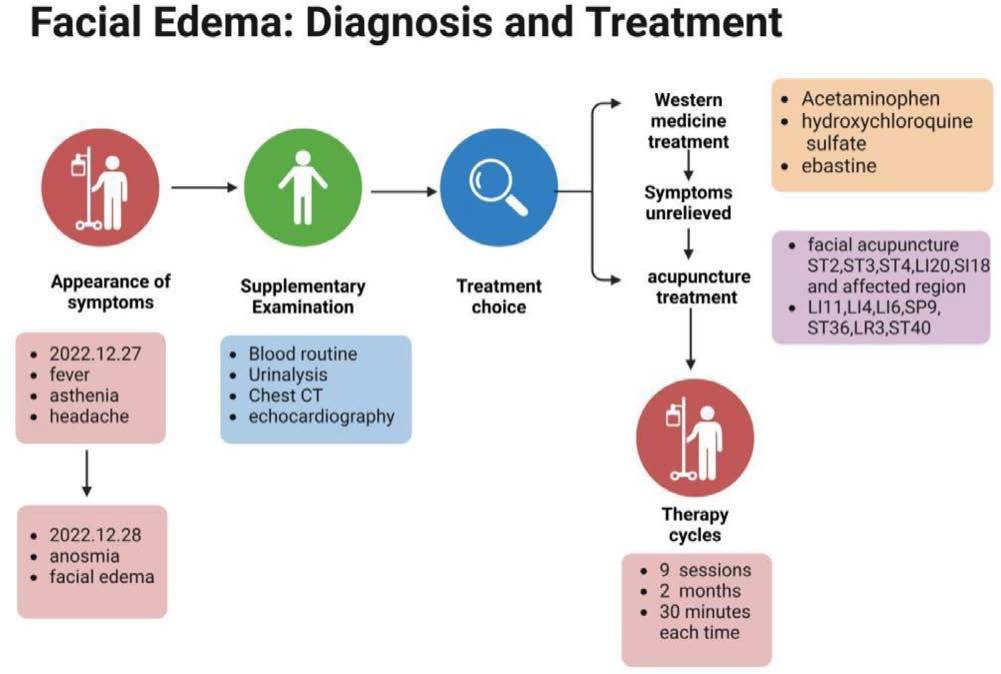
Timeline of the diagnosis and treatment of facial edema.

A 25-year-old female patient was seen at the Acupuncture Clinic of the Third Affiliated Hospital of Zhejiang Chinese Medical University in March 15, 2023, for facial edema persisting for 3 months. Medical history: On December 27, 2022, the patient developed a fever that peaked at 38.7 °C with malaise and headache, and the next day she experienced loss of smell and muscle aches. The patient tested positive for SARS-COV-2 at home using a nasopharyngeal polymerase chain reaction COVID test kit. In addition to the above common major symptoms of COVID-19, the patient developed facial swelling on the second day of fever. The influenza-like symptoms of COVID-19 (fever, malaise, and dyspnea) disappeared on January 3, 2023. However, after 3 months of follow-up, facial edema persisted. Notably, the patient had a background of rosacea, and a history of acne, flushing, and dry skin on the face. She did not present with urinary symptoms. Blood tests, urinalysis, and echocardiography were within normal limits. She was referred to a dermatology department and diagnosed with skin inflammation. After 3 months of aggressive anti-inflammatory treatment with oral hydroxychloroquine and ebastine, the facial edema had not improved. Patient experienced feelings of anxiety, frustration, and desperation due to the change in her appearance. As a result, patient turned to acupuncture treatment (Fig. 1).

The patient developed a fever after SARS-CoV-2 infection. She took oral acetaminophen to reduce the fever and applied an ice pack to her face to physically cool down. On the next day, she developed facial edema, after which she visited the Department of Dermatology, which prescribed the drugs chloroquine bisulfate and ebastin. The symptoms did not relieve after 3-months of Western-drug treatment. Feeling frustrated by the lack of progress, the patient searched online for treatments for facial edema. Upon learning that acupuncture might be effective, she came to acupuncture clinic with a glimmer of hope.

Traditional Chinese medicine believes that according to the symptoms, SARS-CoV-2, can be considered a heat pathogen (toxin, evil) causing dampness, deficiency, and stasis. Heat and inflammation encroached on the facial meridians, leading to poor circulation of qi and blood, further exacerbating the resulting stasis and leading to the occurrence of edema.

Therefore, the key of acupuncture treatment is to remove the pathogenic evil obstructing the channels, transform dampness, dredge the meridians and collaterals, and eliminate edema. In addition, according to our clinical observation, SARS-CoV-2 often attacks the original weak parts of the human body, and induces or worsens the original disease of the patient in the sequelae stage. The patient originally had a history of rosacea. The infection with SARS-CoV-2 may have aggravated her rosacea, leading to facial inflammation and facial edema. The main objectives of acupuncture treatment are to regulate immune function^[15]^ improve local blood circulation and skin metabolism in the face,^[16]^ and alleviate inflammatory response.^[17]^

Based on the above considerations, the acupuncture points included localized acupuncture points on the face (ST2, ST3, ST4, LI20, and SI18), facial acupuncture with multiple micro-needles in the affected area, and distal acupuncture points on the limbs, with acupuncture points on LI11, LI4, and LI6 in the upper limbs, and SP9, ST36, ST40, and LR3 in the lower limbs (Table 1). The patient was in supine position during acupuncture treatment. Before acupuncture, the site of application was sterilized, and needles (diameter 0.18 mm, length 25 mm) were inserted into the above acupoints. The inserted depth was 1 to 3 mm. The needles were retained for 30 minutes. The patient was treated with acupuncture in twenty sessions over 2 months (3 times a week for the first month, 2 times a week for the second month) and instructed not to use any medication during and after the treatment of acupuncture.

**Table 1:** The location of the acupoints used in the patient.

Acupoint	Meridian	Location
Quchi (LI11)	Large intestine meridian of hand-Yangming	Lateral end of the transverse cubital crease, when the elbow is flexed, at the midpoint of the line between Chize (LU5) and the lateral epicondyle of the humerus.
Hegu (LI4)	Large intestine meridian of hand-Yangming	Between the first and second metacarpals, at the midpoint of the radial side of the second metacarpal.
Pianli (LI6)	Large intestine meridian of hand-Yangming	3 inches above the transverse line on the back of the wrist, on the line between Yangxi (LI5) and Quchi (LI11) points.
Yinlingquan (SP9)	Spleen meridian of foot-Taiyin	Medial aspect of the lower leg, in the depression between the lower edge of the medial tibial condyle and the medial edge of the tibial bone.
Zusanli (ST36)	Stomach meridian of foot-Yangming	Anterior aspect of the lower leg on the line connecting Dubi (ST35) with Jiexi (ST41), 3 inches inferior to Dubi (ST35), 1 transverse finger lateral to the anterior tibial crest.
Taichong (LR3)	Liver meridian of foot-Jueyin	Between the 1st and 2nd metatarsals, in the depression anterior to the metatarsophalangeal union, or where the arterial fluctuation is palpable.
Fenglong (ST40)	Stomach meridian of foot-Yangming	Anterolateral aspect of the lower leg, 8 inches above the tip of the ankle, 2 transverse finger-widths from the anterior border of the tibia.
Sibai (ST2)	Stomach meridian of foot-Yangming	Directly below the line of the pupil, in the depression at the infraorbital foramen.
Juliao (ST3)	Stomach meridian of foot-Yangming	Directly below the line of the pupil, at the border of the ala nasi.
Dicang (ST4)	Stomach meridian of foot-Yangming	The Dicang (ST4) point is located on the face, 0.4 inches away from the corner of the mouth.
Yingxiang (LI20)	Large intestine meridian of hand-Yangming	In the nasolabial groove, level with the midpoint of the lateral border of the ala nasi.
Quanliao (SI18)	Small intestine meridian of hand-Taiyang	Directly below the outer canthus of the eye, at the depression of the lower edge of the zygomatic bone.

**Figure 2.**
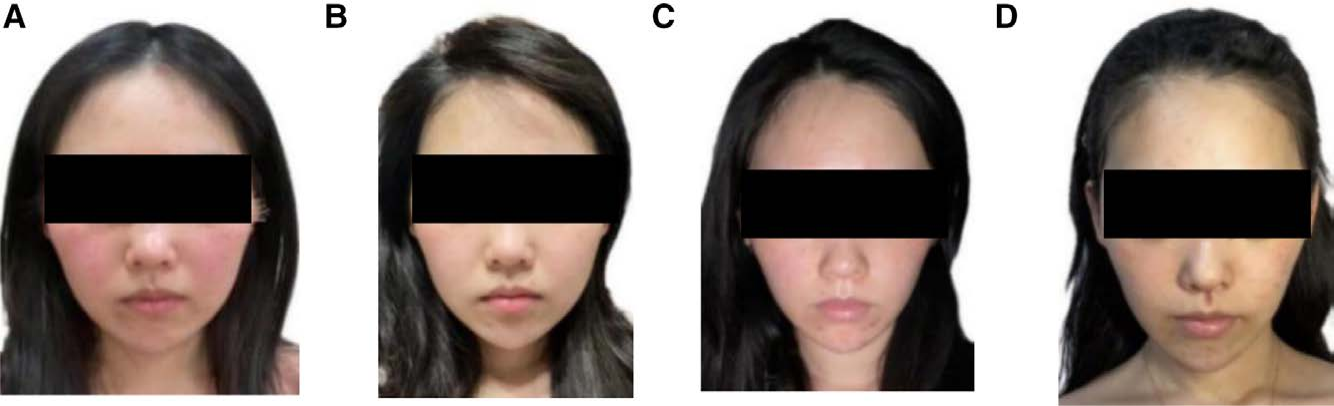
The patient’s appearance before, during, and after acupuncture treatment. (A) Before the acupuncture treatment. (B) One month after acupuncture treatment started. (C) Two months after acupuncture treatment started. (D) Five months after acupuncture treatment.

**Figure 3.**
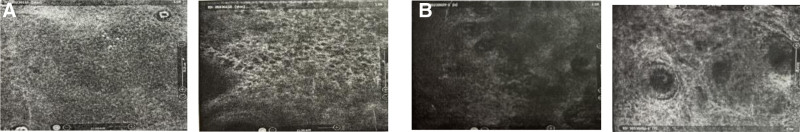
Skin CT before and after treatment. (A) Skin CT on January 18, 2023 suggested that compared to the surrounding normal skin, the epidermis of the lesion is thinner (epidermal thickness of about 30 µm) with mild edema of the stratum spinosum and a small amount of inflammatory cell infiltration. (B) Skin CT on June 9, 2023 suggested that the epidermis is thinner in the lesions than in the surrounding normal skin (epidermal thickness about 37 µm). CT = computed tomography.

**Figure 4.**
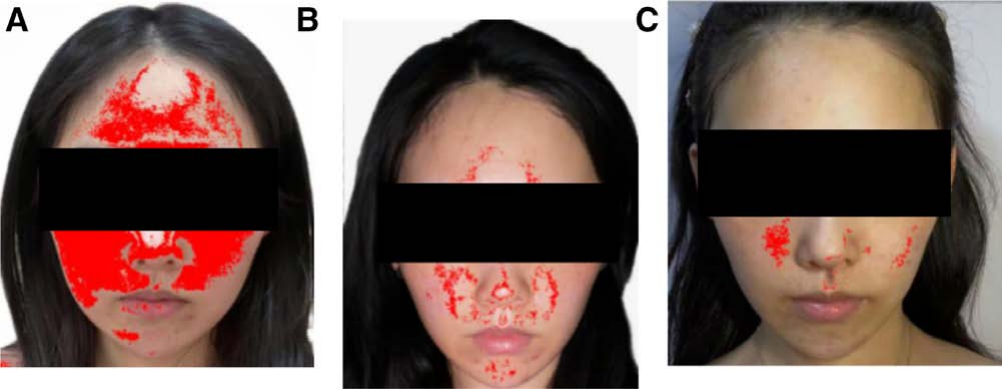
IPP image analysis mapping of facial erythema. (A) Size of the facial erythema before acupuncture treatment. (B) Erythema area 2 months after acupuncture treatment. (C) Erythema area 5 months after acupuncture treatment. IPP = Image-Pro Plus.

Primary outcomes included the patient’s appearance before, during, and after acupuncture treatment (Fig. 2) and the changes in skin computed tomography (CT) (Fig. 3) before and after treatment. Secondary outcomes included the total score of skin lesion score scale, the scores of dermatology life quality index, change in itch intensity of rosacea assessed by Worst Itching Intensity Numeric Rating Scale and the level of emotional state measured by Hamilton Anxiety Scale and Hamilton Depression Scale (Tables 2 and 3), a program called Image-Pro Plus, a 2D image analysis software platform, was used to measure the area of the patient’s facial erythema before and after acupuncture (Fig. 4) and a Patient-Reported Outcomes (Table 4).

**Table 2:** The changes of outcome measures (DLQI, WI-NRS, HAMA, and HAMD) during acupuncture treatments.

	Before treatment	2nd week	4th week	6th week	8th week
	(0 week)				
DLQI (1)	6	5	5	4	3
WI-NRS (2)	3	2	2	2	1
HAMA (3)	25	23	22	21	14
HAMD (4)	20	18	17	14	8

(1) Dermatology Life Quality Index (DLQI): 0–1 no effect at all, 2–5 small effect, 6–10 moderate effect, 11–20 very large effect, 21–30 extremely large effect on patient’s life.

(2) Worst Itching Intensity Numeric Rating Scale (NRS): 0 means no itching, 10 means worst itching imaginable.

(3) Hamilton Anxiety Rating Scale (HAMA): scores below or equal to 17 indicate mild anxiety, 18–24 moderate anxiety, 25–30 moderate to severe anxiety.

(4) Hamilton Rating Depression Scale (HAMD): scoring is based on a 17-item scale. Scores 0–7 normal, 8–16 suggest mild depression, 17–23 moderate depression, 24 + severe depression.

**Table 3:** Skin lesion score scale.

Symptoms	0 point	1 point(slight)	2 point(medium)	3 point (severe)	Before treatment	After treatment
Erythema	None	Flushing (transient erythema)	Flushing of prolonged duration	Nontransient erythema	3	1
Papules and pustules	None	1–10	11–20	≥21	2	1
Telangiectasia	None	Capillary diameter <0.05 mm	Capillary diameter 0.5–1.0 mm	Capillary diameter ≥1.0 mm	2	1
Burning or stinging	None	A little	Often	All the time	3	1
Dry appearance	None	Slight	Medium	Severe	3	1
Plaque	None	Reddish plaque	Red plaque	Dark-Red plaques	2	1
Edema	None	Slight	Medium	Severe	3	0
Ocular manifestations	None	Slight	Medium	Severe	0	0
Total points					18	6

Equation 1 total score = Erythema + Papules and pustules + Telangiectasia + Burning or stinging + Dry appearance + Plaque + Edema + Ocular manifestations.

**Table 4:** Patient-reported outcomes (PROs).

Project	Scoring criteria (0–3 points)	Before treatment	After treatment
Degree of edema	0 = None, 1 = Mild (only perceived swelling), 2 = moderate (visible swelling), 3 = Severe (obvious bulging)	2	0
A sense of tension	0 = None, 1 = Mild, 2 = MODERATE, 3 = Severe	2	0
Indentation upon compression	0 = No depression, 1 = Mild depression (recovery in 2 seconds), 2 = Moderate (recovery in 5 seconds), 3 = Severe (>5 seconds)	2	1
Daily influence	0 = No effect, 1 = Mild discomfort, 2 = Affected expression/chewing, 3 = Severely affected life	1	0
Total score	(0–12 points)	7	1

The primary outcome measures used included skin CT scans at baseline and 6 months later. CT scan results suggested that compared to the surrounding normal skin tissue (50–150 µm), the epidermal thickness of the lesion was thinner (30 µm) with mild edema of the stratum spinosum and a small amount of inflammatory cell infiltration. Skin CT scan after acupuncture treatment showed an improvement in epidermal thickness (about 37 µm), compared to the normal skin tissue (Fig. 3).

The skin lesion score scale was also utilized as a primary measure, calculated by adding 1 to 3 points for each of the following categories, depending on the severity of symptoms experienced (point definition specified in Table 3): erythema, papules and pustules, telangiectasia, burning or stinging, dry appearance, plaque, edema, and ocular manifestations (Table 3). The total Skin Lesion Score reduced from a score of 18 before acupuncture treatment to a score of 6 after acupuncture treatment had concluded.

Using the Image-Pro Plus image analysis software platform, the size of facial erythema declined noticeably after acupuncture treatment and continued to demonstrate improvement at 5-months follow-up (Fig. 4).

## 3. Discussion

SARS-CoV-2 infection triggers the body’s immune response to produce large amounts of inflammatory cytokines, which leads to dry, itchy skin, and exacerbation of the preexisting immune-mediated dermatologic condition rosacea. The mechanism of COVID-19 interacting with the renin angiotensin aldosterone system (RAAS) system stimulates increased vasoconstriction, inflammation, and oxidative stress.^[2]^ “Moreover, chronic activation of RAAS is associated with angiotensin II/angiotensin type 1 receptor deleterious effects, exacerbating the inflammation, fibrosis, apoptosis, antidiuretic hormone, and ultimately water and sodium retention.” The RAAS imbalance may account for some of the patient’s residual long Covid facial edema.^[2]^ Taking anti-inflammatory medications such as hydrochloroquine sulfate and ebastine after developing facial edema did not relieve the symptoms. The use of these allopathic medications can cause side effects in the body. Esther J van Zuuren et al reported a number of side effects for several conventional medical treatments. Minocycline foam 1.5% (FMX103) may have severe side effects such as upper respiratory tract infection, pruritus being the most common cutaneous adverse event. The most common events were dry skin, dry eyes, and dizziness in the hydroxychloroquine group, and dry skin and flatulence in the doxycycline group. However, long-term hydroxychloroquine use can cause irreversible retinopathy, a well-known serious adverse event.^[17]^ After 3 months of treatment with Western medicine, the patient did not show any improvement in facial edema, so she turned to acupuncture.

After 1 month of acupuncture treatment, the facial edema improved substantively. After 2 months of continuous acupuncture treatment (twenty sessions), the patient’s facial edema was nearly gone, and no adverse reactions were observed during the treatment. The patient was very satisfied with the follow-up 3 months after the end of the treatment, and all outcome measurements improved substantially. Why is acupuncture effective in this case? First of all, local acupuncture on the face can help to promote inflammation recovery. The outcome in this case demonstrates the local therapeutic effect of acupuncture. Studies have shown that local modulation of the inflammatory response by acupuncture has clear efficacy and that modulation of the vagus and sympathetic nerves can inhibit the inflammatory response. Yan et al show that through the pathway of somatosensory nerves–vagus nerves-local reflexes, acupuncture can excite the vagus nerves and directly modulate the local inflammatory response of the target organ. Sympathetic activation is synergistically accomplished by the spinal cord and supraspinal nerve centers, with both anti-inflammatory and pro-inflammatory effects, depending on the activated AR. Therefore, acupuncture on distal body acupoints can also exert local anti-inflammatory effects through different reflex pathways. Through the sympathetic–adrenal reflex pathway and somatosensory nerve–sympathetic nerve–local reflex pathway, sympathetic excitability can be inhibited to exert local anti-inflammatory effects.^[18]^ Regarding the mechanisms of acupuncture in alleviating facial edema, conventional perspectives predominantly emphasize its anti-inflammatory effects, while its potential? regulatory functions remain underexplored. Acupuncture stimulation at facial and systemic acupoints modulates ACE2/RAAS imbalance, enhances vascular integrity,^[19,20]^ and orchestrates multi-target, multi-pathway regulation of microcirculatory dysfunction,^[21]^ thereby reinstating lymphovenous hemodynamic equilibrium. This intervention additionally regulates fluid homeostasis-related genes (e.g., AQP3), consequently diminishing interstitial fluid retention and ameliorating tissue edema.^[22,23]^

Secondly, the delivery of healthcare services is embedded within complex psychosocial contexts, which may exert significant influence on treatment outcomes. Alternative therapies such as acupuncture may exhibit clinically significant placebo effects. Although certain therapeutic outcomes of acupuncture could be partially attributable to placebo effects, particularly in managing subjective symptoms, this does not diminish its value as a therapeutic modality. This phenomenon reflects traditional Chinese medicine’s holistic approach to mind–body integration, while simultaneously aligning with contemporary biopsychosocial medical philosophy.^[24,25]^

For patients with facial edema, conventional treatments often include diuretics (e.g., furosemide), antihistamines (for allergy-induced cases), or hormone therapy targeting the underlying causes (e.g., renal or allergic origins). The patient in this case report had previously been treated with oral hydroxychloroquine^[26]^ and ebastine^[27]^ before receiving acupuncture therapy. Conventional treatments may lead to electrolyte imbalances or drug dependence, with targeted limitations that only address symptoms and show limited efficacy for certain functional edemas. The acupuncture treatment we employed reduces edema recurrence by regulating visceral functions (e.g., spleen and kidney), particularly suitable for chronic or idiopathic edema.^[28,29]^ It concurrently improves accompanying symptoms (e.g., anxiety, insomnia) and alleviates virus-related immune-mediated skin lesions by modulating ACE2 receptors or vagus nerve activity. This approach demonstrates comprehensive regulatory advantages for post-COVID complex cutaneous manifestations and can be tailored to individual patient constitutions through syndrome differentiation. It is particularly suitable for chronic or drug-refractory cases. COVID-19-induced inflammation and edema of the facial skin is a new problem, still somewhat uncommon. When encountered in the future, acupuncture treatment presents a safe clinical option according to the principles of local and holistic treatment.

## 4. Limitations

This report is limited to a single-case design, lacking a control group. With a small sample size and individualized interventions, randomization across different conditions or treatment groups was unfeasible, preventing it from replacing group-level randomized controlled trials.^[30]^ These limitations reduce the generalizability of the findings. The absence of standardized acupuncture protocols further limit the study. Future research should include controlled trials with standardized treatment protocols to better evaluate acupuncture’s efficacy and mechanisms in treating post-COVID-19 complications.

## 5. Conclusions

This case report demonstrates the potential efficacy of acupuncture in treating COVID-19-induced facial edema. The patient’s significant improvement after acupuncture, following the failure of conventional treatments, suggests that acupuncture could be a valuable complementary therapy for post-viral inflammatory conditions. Further research through large-scale, randomized controlled trials is needed to validate these findings and clarify the mechanisms involved.

## Acknowledgments

We are grateful for the patient’s trust and for her affirmation of the effectiveness of our treatment.

## Author contributions

**Formal analysis:** Shiqiao He.

**Data curation:** Crystal Lynn Keeler.

**Writing – original draft:** Yike Han

**Writing – review & editing:** Lifang Chen, Hongwu Yin.
